# Photo-addressable microwell devices for rapid functional screening and isolation of pathogen inhibitors from bacterial strain libraries

**DOI:** 10.1063/5.0188270

**Published:** 2024-02-29

**Authors:** Niloy Barua, Ashlee M. Herken, Natalie Melendez-Velador, Thomas G. Platt, Ryan R. Hansen

**Affiliations:** 1 Tim Taylor Department of Chemical Engineering, Kansas State University, 1701A Platt Street, Manhattan, Kansas 66506, USA; 2 Division of Biology, Kansas State University, 1717 Claflin Road, Manhattan, Kansas 66506, USA

## Abstract

Discovery of new strains of bacteria that inhibit pathogen growth can facilitate improvements in biocontrol and probiotic strategies. Traditional, plate-based co-culture approaches that probe microbial interactions can impede this discovery as these methods are inherently low-throughput, labor-intensive, and qualitative. We report a second-generation, photo-addressable microwell device, developed to iteratively screen interactions between candidate biocontrol agents existing in bacterial strain libraries and pathogens under increasing pathogen pressure. Microwells (0.6 pl volume) provide unique co-culture sites between library strains and pathogens at controlled cellular ratios. During sequential screening iterations, library strains are challenged against increasing numbers of pathogens to quantitatively identify microwells containing strains inhibiting the highest numbers of pathogens. Ring-patterned 365 nm light is then used to ablate a photodegradable hydrogel membrane and sequentially release inhibitory strains from the device for recovery. Pathogen inhibition with each recovered strain is validated, followed by whole genome sequencing. To demonstrate the rapid nature of this approach, the device was used to screen a 293-membered biovar 1 agrobacterial strain library for strains inhibitory to the plant pathogen *Agrobacterium tumefaciens* sp. 15955. One iterative screen revealed nine new inhibitory strains. For comparison, plate-based methods did not uncover any inhibitory strains from the library (n = 30 plates). The novel pathogen-challenge screening mode developed here enables rapid selection and recovery of strains that effectively suppress pathogen growth from bacterial strain libraries, expanding this microwell technology platform toward rapid, cost-effective, and scalable screening for probiotics, biocontrol agents, and inhibitory molecules that can protect against known or emerging pathogens.

## I. INTRODUCTION

The application of microbes that are antagonistic toward pathogens has the potential to protect humans, animals, and plants from infection. With respect to crop disease, the application of plant-associated bacteria with known pathogen suppression properties provides a sustainable, environmentally friendly approach to biocontrol that alleviates environmental and health drawbacks associated with synthetic agrochemicals.^1^ As climate change facilitates the emergence of new pathogens and causes difficulty in the management of current crop diseases,^2,3^ the ability to efficiently uncover, modify, and apply new strains of plant-associated bacteria effectively targeted against these pathogens is increasingly valuable. Discovery and characterization can necessitate screening genetically diverse collections of candidate bacteria for those with antagonistic effects on the pathogen. These collections can be taken from a natural environment or mutant libraries generated from a parent strain via transposon mutagenesis. *In silico* identification of antagonistic strains according to genomic information alone is difficult due to the wide range of functional diversity found in many microorganisms and the challenge of predicting a phenotype from a genotype.

Identification of inhibitory microorganisms by direct, phenotypic observation of their interactions with pathogens is an important step in the identification of potential biocontrol agents as it provides insight into the antagonizing organisms' ecological impact on and effectiveness against the competing pathogen.^4^ Traditional, gold-standard approaches for probing microbial interactions use manually paired, dual-species bulk populations on agar plates, an approach that is inherently low-throughput and which typically offers observation of only a small number of interactions (∼10^0^ to 10^1^) between genetically distinct microorganisms per plate.^5^ These experiments are notoriously laborious and slow and typically only provide a qualitative assessment of the interaction. Given the taxonomic and functional diversity found in many microbial communities,^6,7^ as well as the tens of thousands of unique mutations present in mutant libraries, fully exploring interactions in this manner often requires an unfeasible number of plating experiments.^8^

High-throughput screening approaches that offer a quantitative view of microbial interactions at a throughput that matches the scale of genetic diversity in a culture collection, mutant library, or microbial community can rapidly accelerate the identification of candidate biocontrol agents or the identification of genes that impact microbial interactions. Microfluidic devices are particularly useful for probing microbial interactions because they can be designed with precise control of the physical and chemical microenvironments, can track single-cell behavior, and due to their miniaturized nature, provide high-throughput observation. These devices have advanced our understanding of microbial mutualism,^9^ metabolite exchange,^10^ community adaptation to environmental pressures,^11^ and the role of spatial structure in driving community phenotypes,^12^ among other findings. Recently, we reported a photo-addressable microwell device, called the microbial recovery array (MRA), which is a high-throughput, microfabricated screening tool to discover multi-species interaction networks among rhizobacteria and fluorescently labeled focal species.^13,14^ The platform serves as a miniaturized co-culture array, where 10 *μ*m diameter microwells are used to combine the focal species with a unique, randomized combination of unknown bacteria in a confined microscale environment that facilitates intercellular interactions between a small number of cells. The MRA can screen thousands of unique microbial combinations at a time and then recover cells from any well of interest using a novel patterned light extraction system.^15–17^ Recovered cells can then be put into culture so that interaction phenotypes can be confirmed, and the isolated cells can be genetically characterized. This platform was originally developed to screen for interspecies bacterial interactions impacting beneficial plant growth-promoting bacteria (PGPB) to provide insight into interactions within the root microbiome that influence PGPB survival and growth and that lead to successful PGPB colonization in the rhizosphere of various crops, in effort to develop biofertilizer formulations.^13,14^

**FIG. 1.**
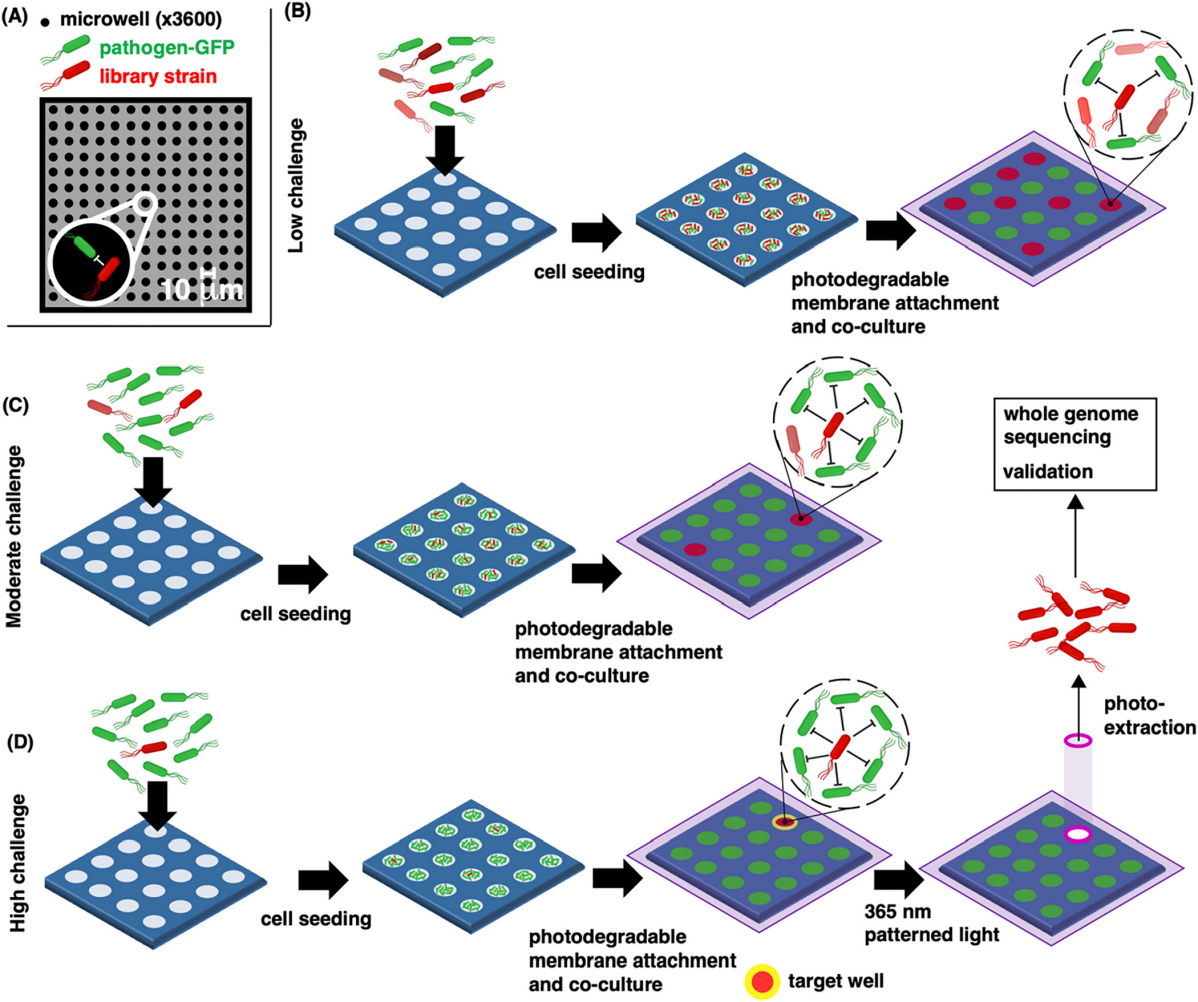
(a) Microwell recovery arrays (MRA) contain a total of 11 025 co-culture sites available to screen a bacteria strain library (here a 293-member collection of biovar 1 agrobacteria recovered from the rhizosphere of *Helianthus annus* plants) for antagonistic interactions with a fluorescently labeled test pathogen (here *A. tumefaciens* sp. 15955 expressing GFP). (b) In the first iteration, unique library strains (denoted by varied shades of red) are combined with the pathogen at an equivalent pathogen-to-strain library cellular ratio, multiple library strains and pathogens may exist in each well. Wells where pathogen growth is significantly diminished are identified as outlier wells (red). (c) Library strains can be cultured against greater numbers of pathogen cells by diluting the strain library into a background of pathogen cells, thereby increasing the pathogen-to-library strain ratio and reducing the number of outlier wells. (d) At a high challenge, library strains are seeded against an excess number of pathogens, enabling the identification of rare outlier wells. These wells contain the strains with the highest levels of pathogen antagonism and are exposed to patterned light to remove a photodegradable polymer membrane, releasing the antagonistic strains from the device for sequencing and further characterization. The figure was prepared with assistance from BioRender software.

In the current work, we develop the MRA for the identification of pathogen inhibitors, expanding its application toward the discovery of new antibiotics, probiotics, and biocontrol agents. We report the first use of the MRA platform in a novel “pathogen challenge mode,” designed to screen a library of candidate biocontrol agents and unveil the strains that display pathogen inhibition under the highest pathogen pressure (Fig. 1). The focal species is a GFP-expressing bacterial pathogen randomly seeded into wells with different strains from the library. Monitoring pathogen growth trajectories in individual wells across the array during co-culture enables high-throughput observation of pathogen–library strain interactions. Wells where growth is significantly diminished are identified as outlier wells, indicating pathogen inhibition. Both the number of bacteria cells and the ratio of different bacteria cells seeded can be controlled by varying the concentration and ratio of cells in the seeding solution, as characterized previously.^18^ Leveraging this capability, sequential iterations of the screen are performed under increasing pathogen-to-library strain cellular ratios [Figs. 1(b)–1(d)]. Individual library strains are, therefore, countered against higher numbers of pathogens with each iteration, reducing the number of outlier wells that display pathogen inhibition. Co-cultures are repeated until outlier wells are rarely observed; at this point, rare outlier wells are identified as target wells [Fig. 1(d)]. This iterative process is designed to control the number of outlier wells generated and to eliminate well-to-well crosstalk and high variance in growth trajectories across the array space, which were significant limitations in prior MRA screens.^13,14^ This enables the clear identification of target wells; these wells contain the strains exhibiting the strongest inhibitory phenotype against the pathogen. Patterned 365 nm light is used to selectively degrade the hydrogel membrane around the perimeter of each individual target well, opening it for strain extraction and recovery. The extraction process is repeated in a sequential manner for each remaining target well to collect further antagonistic strains. Recovered strains are characterized by whole genome sequencing and follow-up assays to validate the pathogen inhibition phenotype.

In the development of this methodology, we chose *Agrobacterium tumefaciens* sp. 15955, a α-proteobacterium of the Rhizobiaceae family that can cause crown gall disease in several plant species,^19^ as the pathogen in the screen. *A. tumefaciens* infects more than 90 families of dicotyledonous plants, resulting in significant agronomic losses.^20–23^ We screen *A. tumefaciens* sp. 15955 against a strain library containing 293 avirulent biovar 1 *Agrobacterium* strains isolated from the roots of *Helianthus annuus* plants. *Agrobacterium* isolates have the potential to be used as biocontrol agents against crown gall disease as they compete for a similar niche in the rhizosphere as *A. tumefaciens* and can produce narrow-spectrum bacteriocins against it. For example, *Rhizobium rhizogenes* K84 (formerly *Agrobacterium radiobacter* K84) and its plasmid-transfer-deficient derivative K1026 have been widely used as crown gall biocontrol agents because of their innate capabilities to produce the bacteriocin, agrocin 84.^24^ K84 was initially isolated from the soil around a plant gall and was the first natural bacterial isolate used as a commercial biocontrol agent.^25^ Despite its success, use of K84 is limited by the specificity of agrocin 84's toxic effects, emphasizing the need for finding a more diverse panel of biocontrol agents against *A. tumefaciens*. Others have uncovered non-pathogenic *Agrobacterium vitis* strains VAR03-1^26^ and E26,^27^ with proven inhibition of agrobacterial pathogens, providing impetus to identify more *Agrobacterium* strains that have the potential for robust protection against crown gall disease.

## II. MATERIALS AND METHODS

### A. Preparation and culture of *A. tumefaciens* strain 15955

Bacterial strains and the plasmids used in this research are listed in Table S1 in the supplementary material, and all genetic manipulations were made using previously described methods.^28^ The plasmid pSRKKm-sfGFP was introduced into *A. tumefaciens* 15955 via mating with *Escherichia coli* S17-1 λ*pir* cells carrying the plasmid. This plasmid was transformed into competent *E. coli* S17-1 λ*pir* cells using calcium chloride heat-shock transformation. *A. tumefaciens* 15955 pSRKKm-sfGFP (herein referred to as 15955-GFP) cells were stored in 25% glycerol at −80 °C. Sterile inoculation loops were used to obtain cells from the frozen stocks to initiate cultures. Cells were cultured in ATGN media [0.079 M KH_2_PO_4_, 0.015 M (NH_4_)_2_SO_4_, 0.6 mM MgSO_4_⋅7H_2_O, 0.06 mM CaCl_2_.2H_2_O, 0.0071 mM MnSO_4_⋅H_2_O, 0.125 M FeSO_4_⋅7H_2_O, 28 mM glucose, pH: 7 ± 0.2] in sterile test tubes supplemented with kanamycin (150 *μ*g/ml) for 24 h (28 °C, 215 rpm).

### B. Extraction and culture of agrobacterial root isolates

Biovar 1 agrobacteria were isolated from the roots of common sunflowers, *Helianthus annuus*, collected at the Konza Prairie Biological Station in northeastern Kansas. Sunflowers were identified as *H. annuus* before blooming based on leaf morphology and plant architecture. Each plant was extracted from the ground using a spade to remove a mass of soil surrounding the upper portion of the plant's root mass. Roots were then gently shaken to remove the soil loosely associated with the plant's roots. Root samples were then placed in a Ziploc bag and put on ice for transport to the lab where samples were weighed, and nine parts sterile, chilled water was added to the Ziploc bag. The bags were agitated every 10 min for 1 h. After this, 100 *μ*l were removed from the soil–water slurry and diluted to recover candidate biovar 1 agrobacteria based on the approach described by Shams *et al*..^29^ The dilutions were plated on media 1A amended with 315 *μ*M tellurite and placed at 28 °C for 3–5 days. Colonies that were round, black, and shiny were then struck onto yeast lactose media and ATGN. After overnight incubation at 28 °C, yeast lactose plates were flooded with Benedict's reagent, and colonies that formed a yellow precipitate were selected from the corresponding ATGN plate for two rounds of streak isolation on ATGN plates. A colony from the second streak plate was then grown in liquid ATGN prior to freezing the cells at −80 °C in 25% glycerol. To combine the library into a single mixture, cultures were initiated using cells from liquid ATGN cultures with an OD_600_ of between 0.4 and 1.0, and library strains were grown individually in ATGN minimal media to mid-log phase. The separate cultures of the biovar 1 *Agrobacterium* library strains were then pooled together at equal volumes, mixed by vortex, and diluted in ATGN to an OD_600_ of 0.1.

### C. MRA design and fabrication

MRAs containing 10 *μ*m diameter microwells, each 20 *μ*m deep and spaced at a 30 *μ*m pitch, were etched on silicon wafers (University Wafers). The array was divided into a 7 × 7 grid of sub-arrays; each sub-array consisted of 15 × 15 arrays of microwells and contained 11 025 microwells available for analysis. Wells were assigned unique on-chip addresses for identification during brightfield microscopy. The detailed layout of the MRA device is described in detail in the supplementary material of Barua *et al.*^13^ The procedure for fabricating the MRA devices is described in Hansen *et al.*^18^ Briefly, 3-in. diameter N-type silicon wafers (University Wafers) were coated with a 1 *μ*m thick layer of Parylene N (PDS 2010 labcoater, Specialty Coating Systems).^30^ Standard photolithography techniques were applied to etch microwells into the wafer. Briefly, wafers were first spin coated with P20 adhesion promoter and then SPR-220 photoresist, both at 300 rpm for 45 s and then baked for 45 s at 115 °C. Wafers were next exposed to UV light for 8 s with a contact mask aligner, hard baked for 45 s at 115 °C, and soaked for 1 min in CD-26 developer solution. Exposed parylene areas on the wafer were then removed with oxygen plasma etching, followed by Bosch etching to fabricate wells at a 20 *μ*m depth. Individual microwell arrays were diced from the wafer using a diamond scriber and stored until further use. Fabrication of the MRAs occurred in a cleanroom environment (Nebraska Nanoscale Facility, University of Nebraska, Lincoln, NE).

### D. Bacteria seeding and trapping in microwell arrays

For preparation of the seeding solutions, a mid-log 15955-GFP culture was diluted to an OD_600_ of 0.1 or concentrated to an OD_600_ of 1 or 10 in ATGN media prior to making seeding solutions. For monoculture studies, the seeding solution contained only 15955-GFP at OD_600_ = 0.1. For co-culture studies, the seeding solution contained 15955-GFP and the biovar 1 *Agrobacterium* strain library mixture (Sec. II B) combined at cellular ratios of 1:1, 10:1, and 100:1. For the 1:1 ratio, OD_600_ = 0.1 suspensions of 15955-GFP and of the strain library mixture were combined at equal volumes. For the 10:1 ratio, OD_600_ = 1 suspensions of 15955-GFP and OD_600_ = 0.1 suspensions of the strain library mixture were combined and diluted to reach an overall cell concentration of OD_600_ = 0.1. For the 100:1 ratio, OD_600_ = 10 suspensions of 15955-GFP and OD_600_ = 0.1 suspensions of the strain library mixture were combined and diluted to reach an overall concentration of OD_600_ = 0.1. These dilutions kept the overall cell concentration at OD_600_ = 0.1, which results in an average cell density of 0.32 cells/*μ*m^2^.^18^ For the 10 *μ*m diameter wells used here, this was equivalent to an average inoculum of ∼25 cells per well. A 700 *μ*l solution of each of these suspensions was incubated on top of microwell arrays for 1 h at room temperature. The parylene layer was then lifted off using Scotch tape to remove cells attached to the background regions of the array.^18^

After cell seeding, photodegradable polyethylene glycol (PEG) hydrogel membranes were attached over the seeded MRA substrates. The membrane was fabricated by first preparing a 49 mM PEG-*o*-ntirobenzyl diacrylate (molecular weight: 3400 Da) solution in ultrapure water and a 20 mM 4-arm PEG-thiol (molecular weight: 10 000 Da) in ultrapure water.^31^ Perfluoralkylated glass coverslip was also prepared through liquid deposition with trichloro(1H, 1H, 2H, 2H,-perfluorooctyl).^16^ Next, the PEG solutions were combined by adding 6.9 *μ*l of the 4-arm PEG solution and 5.6 *μ*l of PEG-*o*-ntirobenzyl diacrylate into 12.5 *μ*l of 1X ATGN buffer (pH 8) to initiate hydrogel formation through base-catalyzed thiol-acrylate Michael addition reactions. Upon mixing, 12.5 *μ*l of the solution was immediately placed on the perfluoralkylated glass coverslip, and 38 *μ*m steel spacers (Precision Brand Products) were placed on the opposing end of the coverslip. The precursor solution was contacted with the seeded microwell array substrate for 25 min to allow for membrane formation and attachment to the microwell substrate. The glass coverslip was then gently removed. This membrane attachment step is described in detail in video format.^15^ After membrane attachment, bacteria are present under the membrane at the bottom of the microwell, and may also be partially embedded within the hydrogel near or in the well.^16^ At this point, the MRA device is ready for co-culture and monitoring with time-lapse fluorescent microscopy (TLFM).

### E. Time-lapse fluorescent microscopy

TLFM images were acquired with a Nikon Eclipse Ti-U inverted microscope equipped with a 20× objective, a motorized XYZ stage, a humidified live-cell incubation chamber (Tokai Hit) with a DS-QiMc monochromatic digital camera, and NIS Elements Image acquisition software. The MRA devices were placed in a custom 3D printed scaffold in order to keep them submerged under liquid media while maintaining a 100 *μ*m distance between the MRA and a base coverslip, as described previously.^13^ Then, the scaffold was placed inside a humidified live-cell incubation chamber at 28 °C during imaging. A FITC filter was used to image 15955-GFP strains (20×, 200 ms, 17.1× gain) with a neutral density filter with 25% standard light intensity to ensure imaging without photobleaching. Brightfield images were also taken at each section of the array after fluorescent imaging. A total of 3,600 of the 11,025 microwells on the array were monitored in the analysis. Images of the microwell arrays were taken every 60 min during culture.

### F. Image analysis

Fluorescent images were used to track 15955-GFP growth during monoculture and co-culture with the strain library and were analyzed using the image analysis protocol described by Timm *et al.*^32^ This procedure uses the Protein Array Analyzer tool in ImageJ to generate growth profiles for each co-culture site, used for identification the top 3 or 4 wells with the lowest growth levels for extraction. First, time-lapse fluorescent images were imported as image sequences corrected by subtracting darkfield images from illumination field images using the image calculator plugin. Then, image backgrounds were removed by selecting a 125-radius sliding paraboloid, and illumination correction was performed using the calculator plus plugin. Finally, 15955-GFP growth in the microwells was calculated using ImageJ “Micro Array” plugin.^32^ Average growth rate and end point fluorescence signals across the 3600 well sub-section of the array with 15955-GFP in monoculture were first determined. End point fluorescence signals and growth rates of 15955-GFP in co-culture at varied library strain to 15955-GFP ratios were next determined. Antagonistic wells were identified as wells showing diminished end point fluorescence signals and growth rates relative to 15955-GFP monoculture baseline levels. Target wells for cell extraction were identified using Grubb's outlier test^33^ by first comparing end point fluorescent signals and then 15955-GFP growth rates. Wells with only lower end point fluorescent signals but with a growth rate similar to the monoculture wells were not included as outliers.

### G. Recovery and labeling of antagonistic library stains from microwell arrays

For the 100:1 15955-GFP:strain library experiments, library strains were extracted from individual target microwells using the Polygon Patterned Illumination Tool (Mightex Systems). Four individual wells (A, B, C, D) were identified as target wells using the image analysis protocol for outlier identification (Sec. II F) and their location on the array was recorded. Each well was then opened in a sequential manner by exposing the well perimeter to 365 nm light patterned in a ring shape (20 *μ*m outer diameter, 10 *μ*m inner diameter). The full procedure for extracting cells out of wells by membrane degradation with patterned UV light was previously described in detail.^15^ After an individual well was opened, it was washed with wash buffer (ATGN media + 0.05% Tween20) to remove cells and the cell suspension was plated onto ATGN-agar media to recover the cells. Two 5 ml washes were performed for each open well. The 10 ml of combined wash solution was then centrifuged (2000 g, 10 min) and cells were resuspended in 2 ml ATGN and plated on ATGN-agar plates. Plates were cultured overnight (28 °C) and individual colonies were then picked from the plate for strain recovery. Four colonies were picked from each plate, and colonies were finally cultured in AT minimal media and stored in glycerol stocks for further validation experiments and for genomic DNA extraction. Antagonistic library strains were labeled according to the well they were extracted from (A–D) and then the colony they were picked from (1–4) during the plating recovery step. For example, library strain A2 designates the strain removed from well A and recovered as the second colony in the plating recovery step. The *Agrobacterium* 3-ketolactose test^29^ was used on recovered strains to quickly verify each was biovar 1 agrobacteria and not an outside contaminating microbe. Strains that failed the 3-ketolactose test were not investigated further.

### H. Follow-up co-cultures to validate antagonistic interactions

After recovery from the MRA device, the interactions between 15955-GFP and the antagonistic library strains recovered from the MRA device were validated using traditional co-culture assays, both in liquid and solid co-culture systems. Liquid co-cultures involved monitoring 15955-GFP growth in cell free culture fluid (CFCF) from each candidate antagonistic strain. To obtain CFCF from an individual strain, each strain was cultured to stationary phase (overnight at 28 °C, 3000 rpm) in 2 ml of ATGN liquid media, and cells were then removed from the media by centrifugation (2000 g, 10 min). CFCF was mixed with 15955-GFP in fresh ATGN media at a 1:1 volumetric ratio to reach an initial OD_600_ value of 0.1 (final volume = 100 *μ*l), at which point 15955 growth was tracked with a Biotek Epoch 2 Multi-Mode Microplate Reader (28 °C, 300 rpm). A monoculture of 15955-GFP was obtained using unconditioned media following the same procedure, here an equivalent volume of 1× PBS was added to fresh ATGN media instead of strain CFCF. As a control to verify that the OD_600_ measurement was due to 15955-GFP growth instead of the growth of possible carryover cells present in the CFCF, CFCF from selected library strains without inoculation of 15955-GFP was also measured. A total of n = 3 independent replicates were measured for each culture condition. The growth kinetics of 15955-GFP in all monoculture and co-culture experiments was analyzed with Growthcurver (R package) to determine the growth rates and carrying capacities of bacteria cells.^34^ Averaged growth metrics of 15955-GFP were calculated for each co-culture and were compared with those from the monoculture using the Wilcoxon two-sample test.

Solid-media co-cultures experiments involved plating 15955-GFP and the antagonistic library strain on ATGN-supplemented agar, where individual library strains recovered from the MRA were spotted in the center of solidified agar loaded with 15955-GFP cells. 15955-GFP and each of the recovered antagonist library strains were first cultured overnight in ATGN liquid media at 28 °C and 215 rpm then diluted to OD_600_ = 0.6 in ATGN media. 35 *μ*l of 15955-GFP were inoculated in tubes containing 10 ml of molten ATGN-agar (65 °C), vortexed vigorously for 10 s, and then poured onto sterile 60 × 15 mm Petri dishes. 7.5 *μ*l of each library strain suspension was then spotted at the center of the solidified agar and allowed to air dry. The plates were then wrapped with parafilm to prevent the shrinking of the media and incubated at 28 °C for 72 h for culture. Plating experiments were repeated n = 3 times. 15955-GFP growth around the central antagonist library strain was monitored, where a zone of growth inhibition serves as a qualitative indicator of antagonism.^35,36^

### I. Whole genome sequencing of antagonistic agrobacterial strains

The Qiagen DNeasy Blood & Tissue Kit (Qiagen, MD) was used to extract genomic DNA from the antagonistic library strains, with minor modifications of the kit protocol as previously described.^31^ DNA concentrations were measured using the Quant-iT PicoGreen dsDNA kit and all samples were normalized to the same concentration. Library preparation for the input agrobacterial strains was done by the Integrated Genomics Facility (IGF) at Kansas State University using Illumina's Nextera DNA Flex kit and Nextera CD Indexes, according to the manufacturer's instructions. Library preparation for recovered agrobacterial cells recovered from the MRA was done by the Microbial Genome Sequencing Center (MiGS; Pittsburgh, PA, USA) using the same kit and reagents as those used by the IGF. In all cases, libraries were sequenced on the NextSeq 550 platform to obtain paired-end reads (2 × 150 bp).

### J. Bioinformatic and phylogenetic analyses

Bioinformatic analyses were performed on Beocat, the High-Performance Computing cluster at Kansas State University. Raw sequences were trimmed using Trimmomatic 0.38^37^ to remove low-quality bases from the 3' end of reads and any adapter sequences. The trimmed short reads were assembled using SKESA 2.4.0^38^ and the resulting contigs were annotated using Prokka 1.13.^39^ Orthofinder 2.5.4^40^ to identify orthologous genes shared across input and recovered strains or shared across recovered strains and select representatives of genomospecies 1 (G1), G2, G3, G4, G5, G7, G8, G9, G13, and G21 [see Fig. 3(b) for specific strains included]. We then used the alignments of single-copy orthogroups generated by Orthofinder as inputs to RAxML 8.2.12^41^ to generate maximum likelihood phylogenetic trees. For these analyses, we used PROTGAMMAAUTO to identify the protein substitution model that best fit each dataset. The raw sequence data is available at the NCBI sequence read archive (BioProject PRJNA 1068816).

## III. RESULTS

### A. MRA screening in pathogen challenge mode

The goal of the MRA screen in pathogen challenge mode was to rapidly evaluate the 293-membered biovar 1 *Agrobacterium* strain library for strains with antagonistic effects on 15955-GFP. In all experiments, bacteria were seeded at an overall (15955-GFP + library strain) OD_600_ of 0.1, but at varied 15955-GFP:library strain ratios (1:1, 10:1, and 100:1). It was essential that seeding solutions in each screening round were kept at a constant overall cell concentration, because growth trajectories are dependent on the total number of cells inoculated in wells,^18^ and needed to be consistent for adequate comparison of arrays under varied pathogen pressure. 15955-GFP growth was directly measured during culture by tracking well fluorescent signals across n = 3600 microwells total and the fluorescent signals of each well were calculated every hour during a 24-h period using ImageJ. Library strains were not fluorescent; thus, only 15955-GFP growth was directly measured.

**FIG. 2.**
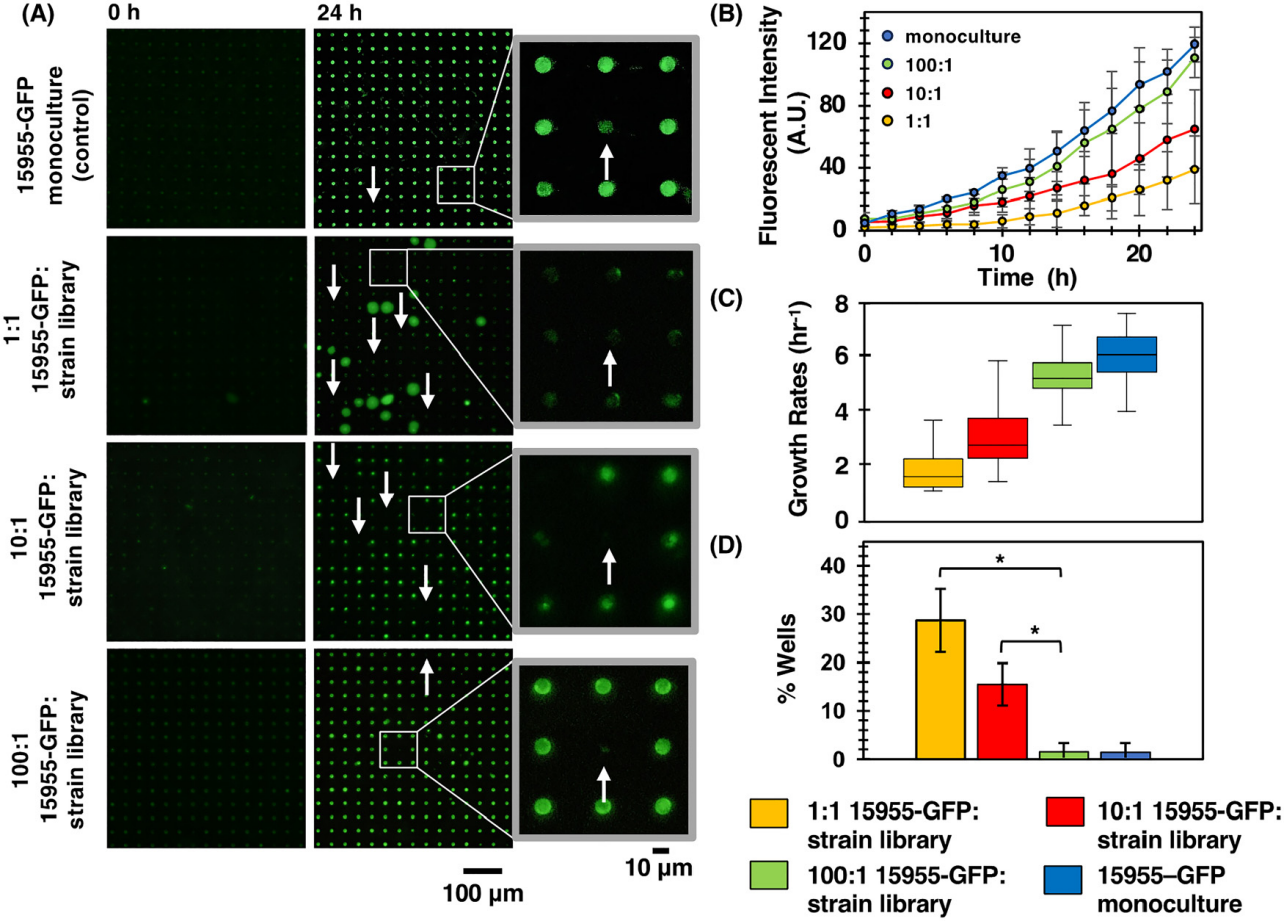
(a) TLFM images of a sample 15 × 15 array of microwells at 0 and 24 h for 15955-GFP monoculture control and 15595-GFP / strain library co-cultures at increasing 15955-GFP to strain library seeding ratios. White arrows denote wells where 15955-GFP growth appeared diminished. (b) Averaged 24h growth trajectories and (c) averaged growth rates from a representative 900 microwell area of the array. (d) Percent of wells with diminished 15955-GFP growth under each culture condition. Wilcoxon’s two-sample tests were conducted to compare the percent of antagonistic wells with the 15955-GFP monoculture. The percent of wells with diminished 15955-GFP growth in the 1:1 and 10:1 co-culture ratios were significantly higher compared to that in the 100:1 co-culture ratio and hence the 15955-GFP monoculture (*, p-value < 0.01). Data in (b)–(d) were generated from screening at each seeding condition n = 4 times.

A necessary first step was to establish a baseline by characterizing 15955-GFP monoculture across the array (Fig. 2**,** 15955-GFP monoculture control), resulting in baseline growth trajectories for comparison with co-culture experiments. Here, 15955-GFP growth is influenced by the physicochemical environment of the microwell but not by interspecies interactions. Cells did not protrude outside of the microwells and into the membrane space above the substrate during culture, as noted in previous MRA experiments,^13^ but instead remained confined within the microwell [Fig. 2(a), monoculture], enabling precise quantification of 15955-GFP growth. Consistent growth trends occurred throughout the array, as a low variance of end point fluorescent levels (σ^2^ = 97) and a nominal growth rate of 6.01 h^−1^ were quantified. A small fraction of wells across the array (1.4%) were identified as outliers due to significantly diminished end point fluorescent signals relative to averaged population levels. As the population of cells seeded in wells follows a Poisson distribution,^18^ it is likely that these outlier wells had lower numbers of 15955-GFP cells inoculated into them, causing cell survival and growth outcomes to become more stochastic.^42^ Alternatively, these wells could contain contaminating microbes that interfered with the monoculture.

Seeding wells with equivalent numbers of *Agrobacterium* library strains and 15955-GFP cells were used as the starting point for co-culture experiments (Fig. 2, 1:1 ratio). Deviations from the control at this 1:1 15955-GFP:strain library seeding ratio verified that library strains input into the device impacted 15955-GFP growth in the screen. Here, variable 15955-GFP growth scenarios were observed after co-culture, evidenced by the overall variance in end point fluorescence levels across the array (σ^2^ = 619), notably higher than the monoculture baseline (σ^2^ = 97). Image analysis identified 11% of wells with enhanced 15955-GFP growth, and 15955-GFP grew out of the well space in some of these wells, appearing as blooms in the fluorescent images. Also, a large fraction of wells (29%) exhibited overall growth rates and end point growth levels that were diminished relative to the monoculture (p < 0.01). The high frequency of wells that deviated from the baseline level after inoculating with a seeding solution of high genetic diversity is a trend that has been noted in other MRA co-cultures,^13,14^ and here may be because multiple library strains were inoculated into each well at this seeding ratio, thus even a few strains from the library capable of impacting 15955-GFP could be seeded into a large fraction of wells across the array. Given the large number of wells putatively showing antagonistic interactions, a second screen was run where library strains were more diluted in the seeding solution by using a 10:1 15955-GFP:strain library ratio (Fig. 2, 10:1 ratio). Here, wells with diminished averaged growth rates and final growth levels across the array were still noted, however, these metrics moved toward the monoculture baseline due to higher numbers of 15955-GFP cells and lower numbers of library strains inoculated into the wells. High variation in end point fluorescent signal was again noted (σ^2^ = 471) and wells with diminished 15955-GFP growth occurred at a moderate frequency (15.5%), still considerably higher than the monoculture baseline. This necessitated the final iteration of the MRA screen where library strains were further diluted in pathogen cells in the seeding solution.

The MRA screen was finally performed using a 100:1 15955-GFP:strain library ratio (Fig. 2, 100:1 ratio). Here, wells were dominated by 15955-GFP cells, rendering conditions similar to the monoculture control. This was evidenced by the averaged 15955-GFP growth rates and final growth levels, which were statistically equivalent to the monoculture control. End point fluorescence signal across the array also had a variance (σ^2^ = 162) closer to the monoculture. However, at this seeding ratio, many wells were inoculated with a small number of library strains within a higher background of 15955-GFP cells. Co-culture between individual strains from the library and an excess number of 15955-GFP cells enabled observation of the most antagonistic interactions, where library strains had to overcome the greatest numbers of 15955-GFP cells that they were paired against. Wells with significantly diminished 15955-GFP growth were detected here, albeit at low frequency (1.5%) equivalent to that of the monoculture control. We hypothesized that these wells contained antagonizing library strains, and the top four outlier wells exhibiting the least amount of 15955-GFP growth were identified as target wells and their location on the array was recorded for library strain extraction.

### B. Photoextraction and identification of antagonistic *Agrobacterium* library strains

**FIG. 3.**
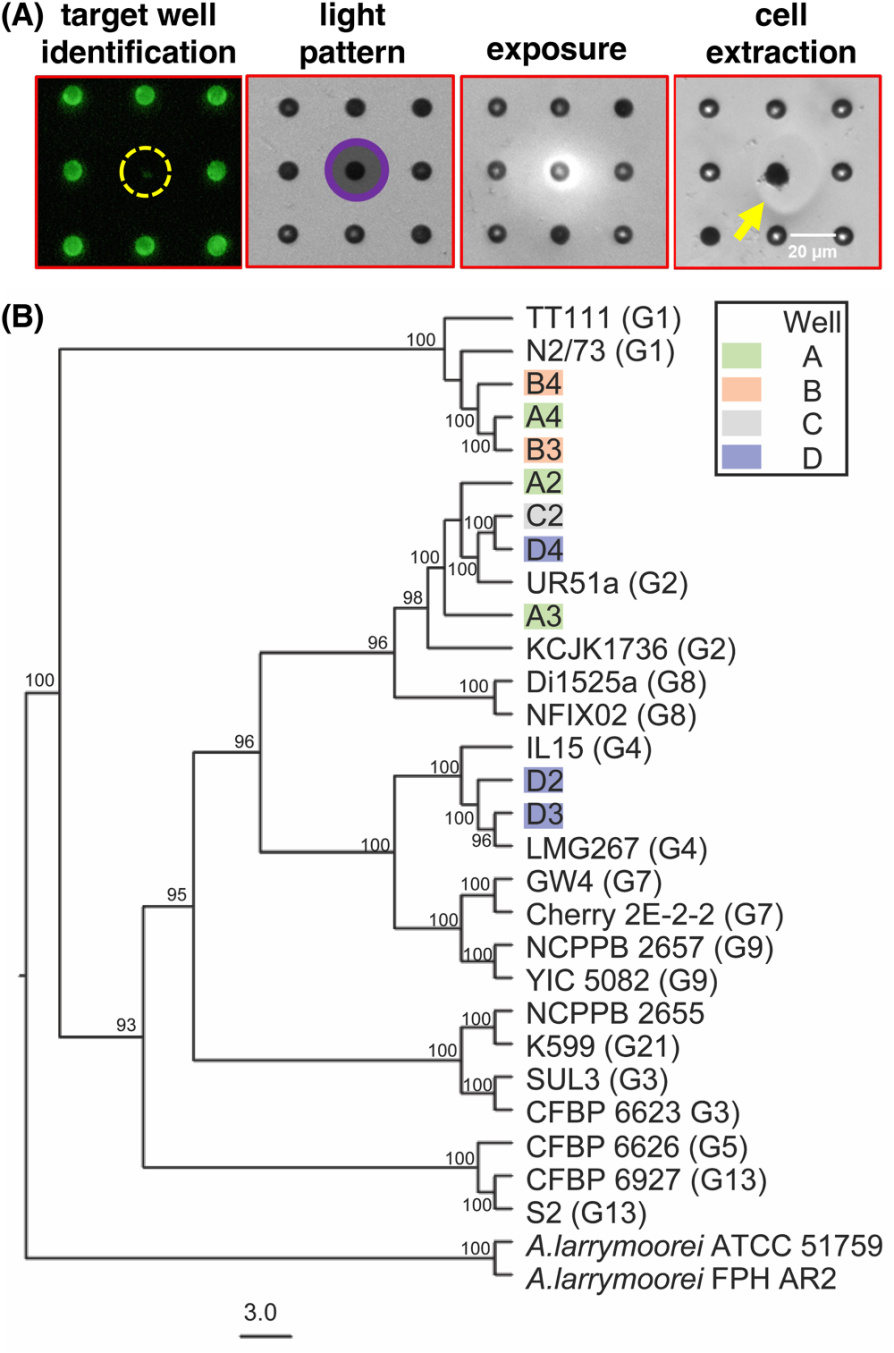
(a) 20× fluorescent and brightfield images of target wells during photoextraction of antagonistic library strains from a target well in the 100:1 15955-GFP:strain library screening step. The yellow arrow highlights the region where the photodegradable membrane was removed during light exposure. (b) Maximum likelihood phylogenetic tree based on 2024 single-copy orthogroups of the recovered agrobacterial library strains (tip labels color-coded based on source well) and select representatives of genomospecies 1 (G1), G2, G3, G4, G5, G7, G8, G9, G13, and G21 using the JTT + F + Γ substitution model and 100 bootstrap replicates. *A. larrymoorei* strain FPH AR2 and strain ATCC 5159 were used as outgroup taxa. Nodes with corresponding bootstrap percentages were labeled and labels were omitted from nodes with less than 90% bootstrap support.

For screening experiments run at the 100:1 15955-GFP:strain library seeding ratio, bacteria cells were extracted from the identified target wells by exposing the well perimeter to 365 nm light (20 mW/cm^2^, 10 min) using the patterned illumination tool. A ring pattern was used to avoid the direct UV exposure of cells within the target wells. These exposure conditions were previously shown to lift off the membrane over target wells, releasing >99.9% of viable bacteria from individual wells into the solution for recovery.^16^ Membrane degradation was confirmed by brightfield microscopy, where a membrane-free area formed exclusively over the target well after UV exposure [Fig. 3(a)]. Cells could be seen moving out of the target well during the extraction step, indicating that bacteria were present in the wells. In the previous characterization of the extraction procedure, it was found that bacteria could be removed sequentially from multiple target wells with minimal cross-contamination, enabling one to open other wells for additional sampling.^16^ Four target wells (wells A–D) were therefore extracted sequentially from a single MRA device. Extracts were plated onto ATGN-agar for strain recovery and further characterization. Four strains were recovered from each well extract, resulting in a total of 16 candidate antagonistic library strains (A1–A4, B1–B4, C1–C4, D1–D4) recovered from the original 293-membered library strain collection that was input into the device.

The MRA screen vastly reduced the number of library strains that were candidates for 15955 inhibition; further characterization of the 16 MRA-selected strains using gold-standard, low-throughput methods now became feasible. The recovered library strains were first qualitatively characterized with the 3-ketolactose test for the ability to convert lactose to 3-ketolactose^29^ to verify they were biovar 1 agrobacteria and not a bacterial contaminant introduced during any MRA processing steps and were then examined in traditional solid-media co-cultures to verify that they again showed antagonism against 15955 (Fig. S1 in the supplementary material). Based on these assays, 11 of the 16 candidates were confirmed as agrobacteria strains antagonistic to 15955, while 5 failed the 3-ketolactose test or co-culture verification test. Subsequent phylogenetic analysis revealed that library strains A4, B3, and B4 were closely related to genomospecies 1 (G1) agrobacterial strains, whereas library strains A2, A3, C2, and D4 were closely related to G2 strains and library strains D2 and D3 phylogenetically clustered with G4 strains [Fig. 3(b)]. Assembly of the sequence reads revealed that samples B2 and C4 were mixed cultures, containing a strain of biovar 1 agrobacteria and a contaminant bacterial strain. These two strains were not investigated further, and the study focused only on the 9 biovar 1 agrobacterial library strains that remained.

### C. Quantitative validation of interactions using liquid co-cultures

**FIG. 4.**
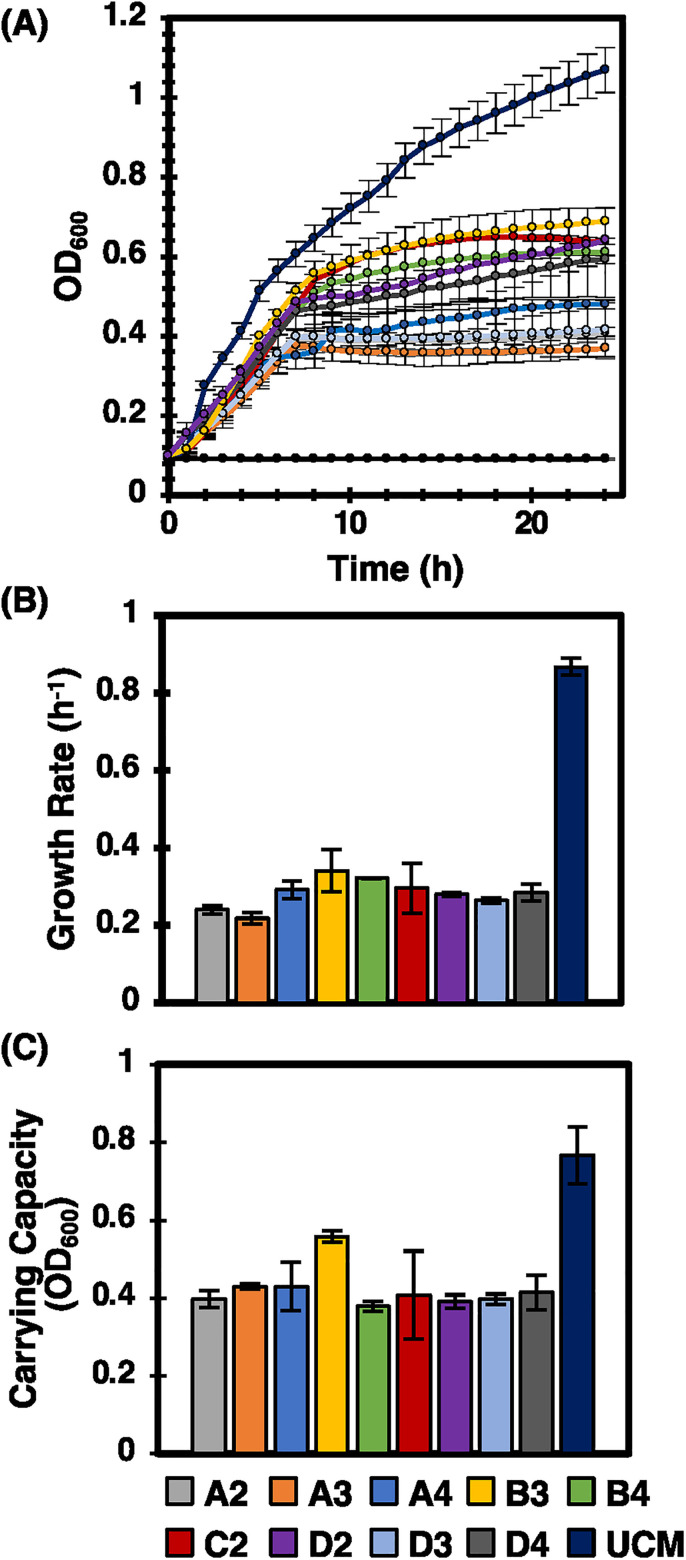
(a) Representative15955-GFP growth trajectories after inoculation into media conditioned by each individual antagonistic library strain recovered from the MRA screen, or into the unconditioned media (UCM) control. Markers represent averaged OD_600_ readings at 30 min time intervals. The black line with no growth represents conditioned media not inoculated with 15955-GFP, which serves as a negative control to verify that no strains or contaminating microbes were present in the conditioned media. (b) 15955-GFP growth rates and (c) carrying capacities corresponding to each growth trajectory. All growth experiments occurred at 28 °C (n = 3 independent experiments). 15955 growth metrics in conditioned media were compared 15955 growth metrics in UCM in (b) and (c), all showed statistically significant differences (Wilcoxon two-sample test, p < 0.01).

To provide quantitative verification of 15955-GFP antagonism from the remaining nine antagonistic library strains uncovered in the MRA screen, 15955-GFP growth was measured during liquid co-culture in media conditioned by each library strain in 96-well plate format. Conditioned media was obtained by combining cell free culture fluid (CFCF) from an antagonistic library strain with ATGN media. 15955 growth trajectories in unconditioned media were measured for comparison. The unconditioned media was ATGN media supplemented with a blank instead of strain CFCF, it therefore contained similar nutrient levels but no strain metabolites. 15955 growth trajectories [Fig. 4(a)], averaged growth rates [Fig. 4(b)], and carrying capacities [Fig. 4(c)] in media conditioned by each antagonistic strain appeared significantly diminished compared to the unconditioned media control (p < 0.01). Conditioned media not inoculated with 15955-GFP was also included as a negative control [black line Fig. 4(a)]. No growth was observed here, which verifies that growth in the other treatments was that of 15955-GFP. These trends quantitatively confirmed that the final nine library strains were inhibitory toward 15955. This finding also provides evidence that 15955 inhibition was diffusive in nature and due to the presence of metabolites (e.g., bacteriocins) secreted in the CFCF, and not from physical cell interactions (e.g., toxin injection into host cell). A diffusive mode of inhibition is highly plausible; well-known examples of *A. tumefaciens* antagonism are caused by inhibitory molecules secreted by related species, including *A. tumefaciens* antagonism by agrocin 84 and secreted by *R. rhizogenes* K84.^24^

### D. Screening outcome

**FIG. 5.**
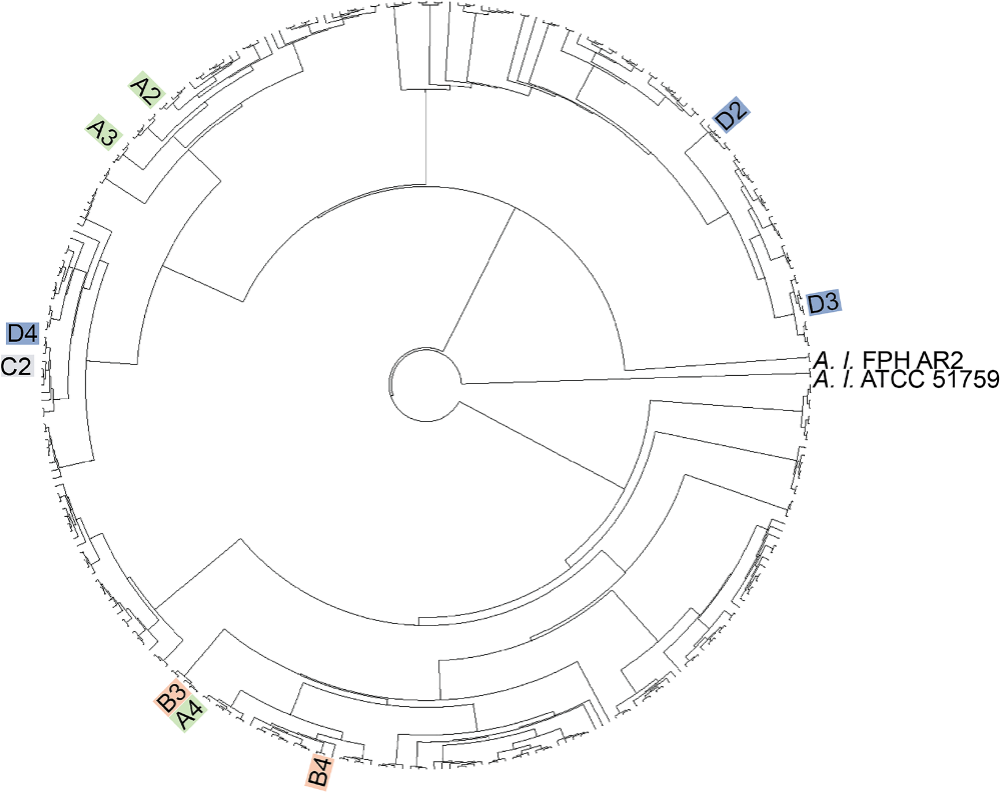
Maximum likelihood phylogenetic tree of 1721 single-copy orthogroups of the nine antagonistic library strains recovered in the MRA screen (tip label colors correspond to the well from which the strain was extracted from) from the 293-strain library input into MRA (unlabeled tips) using the JTT + F + Γ substitution model and 100 bootstrap replicates. *A. larrymoorei* strain FPH AR2 and strain ATCC 5159 were used as outgroup taxa.

A phylogenetic tree that highlights the antagonistic biovar 1 agrobacteria strains identified from the 293-membered strain library is shown (Fig. 5). This illustrates the effectiveness of the MRA device to rapidly uncover antagonistic strains from a genetically diverse strain library. In one iterative screening round, the MRA uncovered 9 novel antagonistic strains from the entire strain library input into the device. To benchmark this result against traditional, plate-based co-culture methods, agar-based pairwise co-cultures were used to test 10 strains randomly selected from the strain library (not screened with the MRA). Like the MRA device, both cell types (15955 and a library strain) are present in the agar plate co-culture, making for a simple comparison between the two approaches. Each strain was tested in triplicate for a total of 30 plating assays, with no trials showing a zone of inhibition; these plates appeared equivalent to mono-cultures control plates in Fig. S1(a) in the supplementary material. This indicates that the randomly selected strains were not antagonists of 15955. The number of plating assays performed here was vastly limited by the time and effort required by this more laborious approach, highlighting a common difficulty encountered when relying on traditional microbiological methods to discover microbe–microbe interactions from functionally diverse culture collections, a limitation that the MRA screening approach directly addresses.

## IV. DISCUSSION

### A. Significance of device

Experimental tools that can rapidly screen heterogenous collections of microbes and identify microbe–microbe interactions of practical importance are currently lacking, a technical limitation that impedes the discovery and development of novel probiotic therapies, bioinoculant formulations, and bioproducts. This is due in large part to the genetic diversity typical of many types of microbiological samples, whether it be a microbial community, a culture collection, or a mutant library of a single species. Other innovative microfluidic systems have been developed for high-throughput analysis of microbial interactions or microbial communities in recent years.^43,44^ Notable droplet microfluidic devices have been developed to screen microbial communities at throughputs ranging from 10^4^ to 10^5^.^45–47^ While such a throughput is not achieved here, a unique feature of the MRA device comes from its photo-addressable nature, enabling the retrieval of live bacteria cells from individual microwells. This addresses a common drawback of droplet microfluidic devices, as many of those devices do not retrieve live cells of interest from the device for downstream analysis or for application of isolated cells, thus limiting analysis to on-chip characterizations. The retrieval capabilities in the MRA enable the use of small diameter wells that partition only a limited number of cells during the seeding step (2–200 cells)^18^ to generate an array of wells that each contain a unique combination of cells for high-throughput screening. Wells provide an attachment surface for bacteria in a spatially confinement environment (10 *μ*m diameter, 0.6 pl volume) to facilitate intercellular interactions at length scales similar to that of a biofilm. For the pathogen inhibition application explored here, the retrieval capability enables follow-up characterization of the interacting cells using a suite of -omic based tools, here whole genome sequencing, and then further development or potential application of the recovered cells, or the inhibitory compounds produced.

The methodology developed here improves the quality of the screen relative to prior MRA reports. A significant difference stems from the use of culturable strain collections, enabling one to run co-cultures in media that accommodate the metabolic demands of every library strain present. Previous efforts involved direct culture of rhizobacteria communities, where observable interactions were vastly limited by culturability (<1% of soil bacteria are culturable)^48^ and were likely biased by the different metabolic growth rates of different community members. By using a pre-cultured collection, a generalist culture media is readily achievable; for the *Agrobacterium* strains investigated here, ATGN media was an obvious choice to minimize metabolic differences between strains that could influence interaction with 15955-GFP. Another important point here is that experimental conditions can be designed to accommodate the size of the library to be screened. With respect to cell seeding conditions, prior characterizations with *E. coli*-GFP indicate that cell seeding events are random and independent and controlled by the concentration of cells in the seeding solution.^18^ With the seeding solution of OD_600_ = 0.1 used here, ∼25 cells on average were inoculated into each well. Thus, by screening 3600 wells, accounting for 9 × 10^4^ cells total, each of the 293 library strains were likely seeded in multiple microwells during the final 100:115955-GFP:strain library screening round. The fact that genetically similar strains were repeatedly recovered from different microwells (B3 and A4; C2 and D4 in Figs. 3 and 5) suggests that the screen was able to effectively uncover 15955 inhibitory strains from the library.

Finally, a significant benefit of the MRA screen comes from its potential scalability to accommodate significantly larger strain libraries. Here, it was only necessary to monitor a 3600 microwell sub-section of the array because only a small library (293 strains) was screened. However, as the array contained 11 025 wells total, the remaining 7425 wells could have been monitored, which would increase the throughput of the screen by threefold to accommodate significantly larger strain libraries. Further increases in throughput could be achieved by designing the array to contain 10^5^ to 10^6^ wells, at which point we expect the screening throughput to become limited by automated image collection using TLFM. Fabrication and materials associated with a single MRA device have been estimated at ∼$15, and a set of three to four devices required for the pathogen challenge assay could be done within a matter of days. The potential savings in time, labor, and material costs increase significantly as higher numbers of strains are included in the screen. While fluorescence assisted cell sorting (FACS) could be a potential alternative method for achieving cell screening and sorting, it is often not directly amenable to co-culture formats, is typically expensive ($100–200/h), and is usually confined to core research facilities. Thus, the MRA screen holds unique potential for application in this context.

### B. Future device optimization and future applications

The results highlight the capabilities and benefits of the MRA challenge-mode screen but also identify its current limitations. It should be noted that a significant portion of strains recovered from the screen (7/16) did not show antagonistic interactions toward 15955-GFP in follow-up assays and that outliers microwells were detected in monoculture control experiments [Fig. 2(d)]. Both findings highlight the possibility of false positives, a limitation that was remedied by extracting cells from multiple outlier wells in the final screening iteration to increase the number of candidate strains for follow-up analysis. False positives could be reduced by screening strain libraries with antibiotic resistance when possible, or by integrating the MRA substrates into a microfluidic device for automated sample handling, both of which reduce the likelihood of contaminating microbes being recovered from the device. Another current limitation of the device is that it does not provide information on the nature of the inhibitory interaction; physical interactions appear similar to diffusive interactions, as both inhibit pathogen growth. The use of focal species engineered to produce a fluorescent marker reporting a specific interaction (GFP expression on quorum activation^49^ or type VI secretion,^50^ as examples), would provide higher biological specificity during the screen. Finally, further procedural steps could be taken to optimize the cell survivability and sensitivity of the method. It is possible that a fraction of cells seeded did not survive the MRA processing steps, which means that some inhibitory strains from the library could be missed during the screen. Further platform optimization to maximize cell viability through MRA processing steps will ensure that all strains are observed during the screen.

It is expected that the optimized MRA methodology can also be used to screen interactions across large mutant libraries, an effort necessary to connect a microorganisms' interaction phenotype (e.g., antagonism) with its genotype. Similar to the samples analyzed here, a transposon mutant library would generate a genetically diverse strain mixture from a parent strain. For bacteria with large genomes, such as *Streptomyces* species (genome size = 8.7–11.9 Mbps)^51^, screening a mutant library to saturation would require observation of the interaction of ∼60 000 mutants with the pathogen, while the *Agrobacterium* species used here (genome size 5.67 Mbps)^52^ would require observation of ∼28 000 mutants. Such a mutant library could be generated from an inhibitory *Agrobacterium* strain found in this study and then combined with 15955-GFP in the MRA device. Screening for unrestricted growth of 15955-GFP across the MRA would identify microwells containing a mutant with loss of inhibitory function. Subsequent extraction and transposon mapping of this mutant strain would identify the genes responsible for antagonism, which could lead to the discovery of new inhibitory molecules (i.e., bacteriocins) and/or an understanding of the mechanisms responsible for inhibition. While accommodating these numbers of strains with traditional plate-based assays would be infeasible, future efforts to scale up the throughput of the MRA to meet these higher screening requirements will enable a rapid screen of interactions within a mutant library to saturation. In addition to mutant libraries, researchers continue to build large culture collections of plant-associated bacterial isolates that require screening for interactions against plant pathogens. For example, Carper *et al.* recently reported 3211 bacterial isolate library across 120 genera from the *Populus* root microbiome, including previously unknown species.^53^ With the variety of antagonistic microorganisms present in the rhizosphere and endosphere,^54^ additional screening across these types of large culture collections is likely to uncover novel isolates, gene clusters, and natural products with potential use in biocontrol. Beyond agriculture, rapidly uncovering cellular inhibitors of pathogens is relevant to human and animal health by identifying strains that can be used for probiotic manipulation of human and animal gut microbiota^8^ and for identifying new strains and compounds that can combat antibiotic resistant pathogens.^55^ The foundational MRA screening methodology established here extends directly to these high-priority applications.

## V. CONCLUSIONS

The high-throughput screening of *A. tumefaciens* 15955-GFP interactions across a strain library of biovar 1 *Agrobacterium* strains taken from *H. annuus* roots was demonstrated by operating the MRA in the pathogen challenge mode. Key steps in the procedure involved first characterizing 15955-GFP monoculture growth in the MRA, then seeding MRAs with increasing 15955-GFP:strain library ratios until growth trajectories across the array were similar to the monoculture control, thereby allowing outlier microwells containing strains with the most antagonistic interactions toward 15955-GFP to be identified. After extraction of antagonistic library strains from these wells using a patterned light extraction system, whole genome sequencing analysis revealed the identity of each strain, and follow-up assays revealed that 15955-GFP growth inhibition was diffusive in nature. Identification of bacteriocin gene clusters and further mechanistic evaluation of the inhibitory interactions is the subject of future investigation. This progress lays the foundation for high-throughput screening of interactions between pathogens and large culture collections of plant-associated bacteria to uncover new cellular inhibitors with potential use for protection against plant disease. The MRA pathogen challenge screening methodology has a broader impact on applications related to human and animal health, such as the discovery of therapeutic microbes and bacteriocins narrowly targeted against pathogens from the gut microbiome,^55^ where similarly high demands in screening throughput exist.

## SUPPLEMENTARY MATERIAL

The supplementary material file contains a table of all bacterial strains and plasmids used in this study (Table S1) and a sample agar co-culture plating assay used to verify antagonism between *A. tumefaciens* sp. 15955 and antagonistic library strains isolated from this screen (Fig. S1). A portion of this manuscript is available as an academic thesis dissertation.^56^

## Data Availability

The data that support the findings of this study are available from the corresponding author upon reasonable request.
